# Identification of tRNA‐derived small noncoding RNAs as potential biomarkers for prediction of recurrence in triple‐negative breast cancer

**DOI:** 10.1002/cam4.1761

**Published:** 2018-09-21

**Authors:** Wanting Feng, Yongfei Li, Jiahui Chu, Jun Li, Yanhong Zhang, Xiaorong Ding, Ziyi Fu, Wei Li, Xiang Huang, Yongmei Yin

**Affiliations:** ^1^ Department of Oncology The First Affiliated Hospital of Nanjing Medical University Nanjing China; ^2^ Department of Oncology The Affiliated Huaian NO. 1 People's Hospital of Nanjing Medical University Huaian China; ^3^ Department of Pulmonary and Critical Care Medicine Fuzhou General Hospital Fuzhou China; ^4^ Nanjing Maternal and Child Health Medical Institute Obstetrics and Gynecology Hospital Affiliated to Nanjing Medical University Nanjing China

**Keywords:** biomarker, stem cells, tDRs, triple‐negative breast cancer

## Abstract

Triple‐negative breast cancer (TNBC), an intrinsic subtype of breast cancer, is characterized by aggressive pathology and shorter overall survival. Yet there is no effective therapy for these patients. Breast cancer stem cells (BCSCs) in TNBC may account for treatment failure. It is urgent to identify new therapeutic targets for TNBC. tRNA‐derived small noncoding RNAs (tDRs) represent a new class of small noncoding RNAs (sncRNA), which have been reported in some human diseases and biological processes. However, there is no detailed information about the relationship between tDRs and BCSCs. In this study, a population of CD44^+^/CD24^−/low^ cells was isolated and identified by reliable BCSC surface markers. tDR expression profiles in TNBC and non‐TNBC CSCs were performed by RNA sequencing. A total of 1327 differentially expressed tDRs contained 18 upregulated and 54 downregulated in TNBC group. Furthermore, the selected tDRs were validated by quantitative reverse transcription‐polymerase chain reaction (qRT‐PCR). tDR‐000620 expression level was consistently lower in TNBC cell lines CSCs (all *P *<* *0.05) and serum samples (*t *=* *2.597, *P *=* *0.013). tDR‐000620 expression was significant association with age (*P *=* *0.018), node status (*P *=* *0.026) and local recurrence (*P *=* *0.019) by chi‐square test. Kaplan‐Meier method with log‐rank test for comparison of recurrence curves. The results showed that the tDR‐000620 expression (*P *=* *0.002) and node status (*P *=* *0.001) group were statistically significant with recurrence‐free survival. Furthermore, multivariate Cox regression demonstrated that lymphatic metastasis (HR, 3.616; 95% CI, 1.234‐10.596; *P *=* *0.019) and low tDR‐000620 expression (HR, 0.265; 95% CI, 0.073‐0.959; *P *=* *0.043) were two independent adverse predictive factors for recurrence‐free survival. Finally, we found that tDR‐000620 participated in some important biological processes though GO and KEGG analysis. Taken together, our study reveals the expression profiles of tDRs in TNBC and non‐TNBC CSCs. It offers helpful information to understand the tDR‐000620 expression is responsible for the aggressive phenotype of BCSCs. It may provide predictive biomarkers and therapeutic targets for TNBC recurrence.

## 1 INTRODUCTION

Breast cancer is one of the most widespread carcinoma and the main causes of cancer‐related death in women. An increasing trend of incidence and mortality rate is observed among women in recent years.^1^ Triple‐negative breast cancer (TNBC) does not express estrogen receptor (ER), progesterone receptor (PR), and human epidermal growth factor receptor 2 (Her‐2) gene, resulting in aggressive pathology, early peak of recurrence, and shorter overall survival than other subtypes. The lack of endocrine therapy and Her‐2 targeted agents; standard chemotherapy is the main method of systemic treatments.^2^, ^3^, ^4^ Therefore, it is necessary to investigate TNBC aggressive pathology mechanism and the potential therapeutic targets.

The CSC theory^5^, ^6^ may cast a new direction for TNBC therapy recent years. CSCs have been associated with normal stem cell‐like characteristics, including self‐renewal, differentiation, enhanced proliferative and invasive properties, chemoradiation resistance, high tumorigenic feature, and so on.^7^ A population of CD44^+^/CD24^−/low^ cells has been demonstrated to have tumor‐initiating properties in breast cancer in 2003.^8^ The accumulating evidences suggested that breast cancers were initiated and maintained by a part of tumor cells with stem cell properties. As stated above, we hypothesize that the existence of CSCs might be the main cause for the extensive proliferation, invasion, metastasis, and resistance of TNBC. In order to improve survival and quality of life of breast cancer patients, it is certainly worth digging further the potential mechanism of maintain this tumor‐initiating ability and CSC‐targeted therapy.

Transfer RNA (tRNA) is traditionally considered as a key player of the protein synthesis machinery and post‐transcriptionally modified.^9^ tRNA halves (tiRNAs) and tRNA‐derived fragments (tRFs) represent a novel class of tRNA‐derived small noncoding RNAs, which are involved in several biological processes some human diseases with the development of high‐throughput sequencing.^10^, ^11^, ^12^ Growing studies have demonstrated that the tRNA fragments could derive from precise cleavage of tRNAs and not random degradation products. If cleavage sites are in the anticodon loop under stress conditions, these fragments are called tiRNAs (29‐50 nucleotides), which include 5′‐tRNA halves (tiRNA‐5) and 3′‐tRNA halves (tiRNA‐3).^13^ According to their sites of sequence in pre‐tRNA or mature tRNA, tRFs (16‐28 nucleotides) can be separated into three subtypes: tRF‐5 series, tRF‐3 series, and tRF‐1 series,^14^ while, another recently characterized tRFs are those derived from the internal region of the mature tRNA sequence, termed internal tRFs(i‐tRF).^15^

The investigation of tRNA fragments is a emerging field in recent years. Actually, many studies have been showed that aberrant expression level of tRFs&tiRNAs was participated in gene expression, biological processes, tumorigenesis, innate immunity, some diseases, and served as biomarkers. The research from Lee YS found that tRF‐1001, a 3′tRF derived from pre‐tRNA‐Ser, was highly expressed in different cancer cell lines. And the expression of tRF‐1001 was firmly correlated with cell proliferation, and knockdown tRF‐1001 can inhibit proliferation with the accumulation of cells in G2 phase and DNA synthesis in prostate cancer.^12^ In another study, tRFs can compete for the mRNA binding sites of YBX1 to suppress cancer cell growth and invasion.^16^ In addition,two tRFs derived from tRNA^Lys‐CTT^ and tRNA^Phe‐GAA^ may serve as good indicators of PFS and prognostic markers.^17^ Moreover, a number of neurological disorders were caused by defects in tRNA metabolism and tRNA processing enzymes.^10^ As previously mentioned, these studies strongly indicated that tDRs may serve as a basis for intensive study on the potential biomarkers and therapeutic targets. However, studies based on noncoding RNA expression profiles of cancer stem cells are limited^18^, ^19^ and the role of tDRs in TNBC CSCs is still missing.

In order to uncover potential mechanism of maintain stem cell ability in TNBC and the value of prediction biomarkers, we applied immunomagnetic beads to sort a subpopulation of CD44^+^/CD24^−/low^ cells from TNBC cell line MDA‐MB‐231 and non‐TNBC cell line MCF‐7. And then we verified their CSC properties in vitro conditions. Moreover, tDRs expression profile analysis was performed by RNA sequencing to further explore the different expression of tRFs&tiRNAs, and then identified which tDRs might be potential biomarkers for prediction of recurrence in triple‐negative breast cancer.

## 2 MATERIALS AND METHODS

### 2.1 Patient samples

Totally, 44 primary TNBC blood samples and 28 primary non‐TNBC blood samples were obtained from breast cancer patients who had histologically confirmed after informed consent at the First Affiliated Hospital of Nanjing Medical University (Nanjing, China) between 1 June 2014 and 31 August 2015. The latest follow‐up was updated in April 2018. Main clinical endpoint was recurrence‐free survival. The blood samples were collected in BD Vacutainer plastic serum separator tubes (SSTs; catalog no. 367985; BD Biosciences, San Jose, CA, US). The samples centrifuged at 1200 *g* for 10 minutes after incubating at room temperature for 15 minutes. The serum supernatant was transferred into new tubes and centrifuged at 16 000 g for 15 minutes to remove the residual cells and debris. Finally, the serum samples were aliquoted to new tubes and stored at −80°C until further processing. This study was approved by the First Affiliated Hospital of Nanjing Medical Ethics Committee.

### 2.2 Cell lines and cell culture

The human non‐TNBC cell line MCF‐7, T47D, and SKBR3 and TNBC cell line MDA‐MB‐231, HCC‐1937, and MDA‐MB‐453 were originally imported from Cell Bank of Chinese Academy of Sciences (Shanghai, China). MCF‐7, T47D, SKBR3, and HCC‐1937 were maintained in DMEM (Gibco, CA, USA) supplemented with 10% (0.1 g/mL) fetal bovine serum (FBS, Gibco, CA, USA) 100 U/mL penicillin and 100 μg/mL streptomycin (Gibco, CA, USA). They were grown in humidified incubator with 5% CO_2_ at 37°C. And MDA‐MB‐231 along with MDA‐MB‐453 was cultured in L15 (Gibco, CA, USA) without 5% CO_2_.

### 2.3 Breast cancer stem cell fractionation

Breast cancer stem cell isolation was performed as previously described.^20^ The breast cancer cells were rinsed with PBS with 2% FBS and suspended in PBS containing 0.5% FBS and PI (0.5 mg/mL). CD44 and CD24 microbeads were applied for sorting cell lines into positive and negative fractions on MACS column (Miltenyi Biotec, Bergisch Gladbach, Germany). CD44^+^/CD24^−/low^ cells were cultured in serum‐free DMEM/F12 medium (Gibco, CA, US).

### 2.4 Sphere formation assays

CSCs from non‐TNBC and TNBC cell lines were cultured in six‐well ultralow attachment surface plates (Corning, Acton, MA, USA). All BCSCs were cultured at a density of 5000 cells per well in serum‐free DMEM/F12 medium, supplemented with 20 ng/mL recombinant epidermal growth factor (EGF), 10 ng/mL basic fibroblast growth factor (bFGF), and 5 mg/mL insulin (Sigma, St. Louis, Missouri, USA). BCSCs were incubated at 37°C in a humidified atmosphere with 5% CO_2_. The number of spheres for each well was evaluated after 7 days of culture under the light microscope. Round cell clusters >50 μm were judged as spheres.

### 2.5 Western blot

Breast stem cells were prepared using RIPA lysis buffer, and protein content was tested by bicinchoninic acid kit (Beyotime, Shanghai, China). Total proteins were separated by 10% SDS‐PAGE and transferred to polyvinylidene difluoride membranes. The membranes were blocked with 5% nonfat milk at 37°C for 1 hour after two washes with TBST. Then, the membranes were incubated with anti‐ALDH1 (1:1500 rabbit), anti‐Oct4 (1:1500 rabbit), anti‐Sox2 (1:2000 rabbit), and anti‐GAPDH (1:3000 rabbit) antibodies (Abcam, Shanghai, China) at 4°C over night. After two washing, the membranes were incubated with secondary antibody conjugated to HRP (1:5000) at 37°C for 1 hour. GAPDH was used as a loading control.

### 2.6 RNA isolation

Total RNA was extracted from clinical serum samples and stem cells using TRIzol^™^LS and TRIzol^™^ reagent, respectively, according to the manufacturer's protocol (Invitrogen, CA, USA). RNA concentration and purity from each sample were quantified using a NanoDrop ND‐1000 instrument. Approximately 200 ng of total RNA from each sample was used for high‐throughput sequencing.

### 2.7 tRFs&tiRNAs sequencing data analysis

Total RNA of each sample needs to be removed some RNA modifications that interfere with small RNA‐seq library construction. Subsequently, total RNA was ligated to 3′ and 5′ small RNA adapters for tRFs&tiRNAs‐seq library preparation. And then, cDNA was synthesized and amplified using RT and amplification primers (Illumina, San Diego, CA, USA). Finally, the tRFs&tiRNAs‐seq libraries were quantified by Agilent Bioanalyzer 2100 and sequenced using Illumina NextSeq 500 system (#FC‐404‐2005, Illumina, San Diego, CA, USA) according to the manufacturer's instructions.

### 2.8 GO and pathway analyses

Gene ontology (GO) provides a controlled vocabulary to describe gene and gene product attributes (http://www.geneontology.org/).^21^ GO covers three parts: cellular component, biological process, and molecular function. The *P*‐value denotes the significance of GO term enrichment in the differentially expressed tRFs&tiRNAs list, and false discovery rate (FDR) was applied to correct the *P*‐values by the method described by Benjamini and Hochberg.^22^ The lower the *P*‐value, the more significant the GO term (*P*‐value <= 0.05 is recommended). Pathway analysis was based on the Kyoto Encyclopedia of Genes and Genomes (KEGG) database (http://www.kegg.jp/) which was used to describe intracellular pathways with significant differentially expressed tRFs&tiRNAs. The *P*‐value (EASE‐score, Fisher‐*P* value, or Hypergeometric‐*P* value) denotes the significance of the pathway correlated with the conditions. Lower the *P*‐value, more significant is the pathway. (The recommend *P*‐value cutoff is 0.05).

### 2.9 Validation of differentially expressed tDRs by qRT‐PCR

Total RNA from BCSCs and clinical serum samples was pretreated with rtStar^™^ tRFs&tiRNAs Pretreatment Kit (Arraystar, Rockville, MD, USA) to remove modifications. Then, the RNA was reverse transcribed into cDNA and PCR amplifications were performed using Bulge‐Loop^™^ miRNA qRT‐PCR Starter Kit (Ribobio, GuangZhou, China) following the manufacturer's instruction. The reaction mixture of samples was initially incubated for 10 minutes at 95°C and then incubated at 95°C for 15 seconds, 60°C for 1 second, 72°C for 30 seconds for 35 PCR cycles in the Applied Biosystems 7900 Real‐Time PCR System (Applied Biosystems, Foster City, CA, USA). Signals were detected at the end of each cycle. The informations about tDRs are list in Table 1, including names, sequences, and the specific primer sequences for PCR.

**Table 1:** Sequence of primers of tDRs used for qRT‐PCR

Primer name	Sequence	
tDR‐001262	RT	GTCGTATCCAGTGCGTGTCGTGGAGTCGGCAATTGC ACTGGATACGACAGAGCGCC
	Forward	ATAATAATCCCTGGTGGTCTAGTGGTTAGGATTC
tDR‐001267	RT	GTCGTATCCAGTGCGTGTCGTGGAGTCGGCAATTGC ACTGGATACGACAACCAGG
	Forward	ATTATTCCCACATGGTCTAGCGGTTAGGATT
tDR‐008406	RT	GTCGTATCCAGTGCGTGTCGTGGAGTCGGCAATTGC ACTGGATACGACCCAGTAC
	Forward	AGCTGCGTTCGATCCCC
tDR‐000620	RT	GTCGTATCCAGTGCGTGTCGTGGAGTCGGCAATTGC ACTGGATACGACCGCCGAAT
	Forward	ATTATATCCCTGGTGGTCTAGTGGCTAGG
tDR‐000106	RT	GTCGTATCCAGTGCGTGTCGTGGAGTCGGCAATTGC ACTGGATACGACTGGTGC
	Forward	AGCTCACCCGGGTTTCG
tDR‐007257	RT	GTCGTATCCAGTGCGTGTCGTGGAGTCGGCAATTGC ACTGGATACGACCGAACC
	Forward	GCAGCGGAAGATCGCG

### 2.10 Statistical analysis

Data were conducted using SPSS software (SPSS 22.0, Chicago, IL, USA). Data are expressed as means ± standard deviation (SD). The *P*‐values <0.05 were considered statistically significant. The difference of tDRs levels between two groups was measured with unpaired *t* tests. The correlation between tDRs expression and clinicopathological features was analyzed by Pearson chi‐squared test and Fisher's exact test. Kaplan‐Meier method was used to calculate recurrence and metastasis rate, and log‐rank test was carried out to compare rate of each group. Multivariate Cox proportional hazard model was used to analyze different variables related to recurrence‐free survival.

## 3 RESULTS

### 3.1 Isolation and characterization of BCSCs

**Figure 1:**
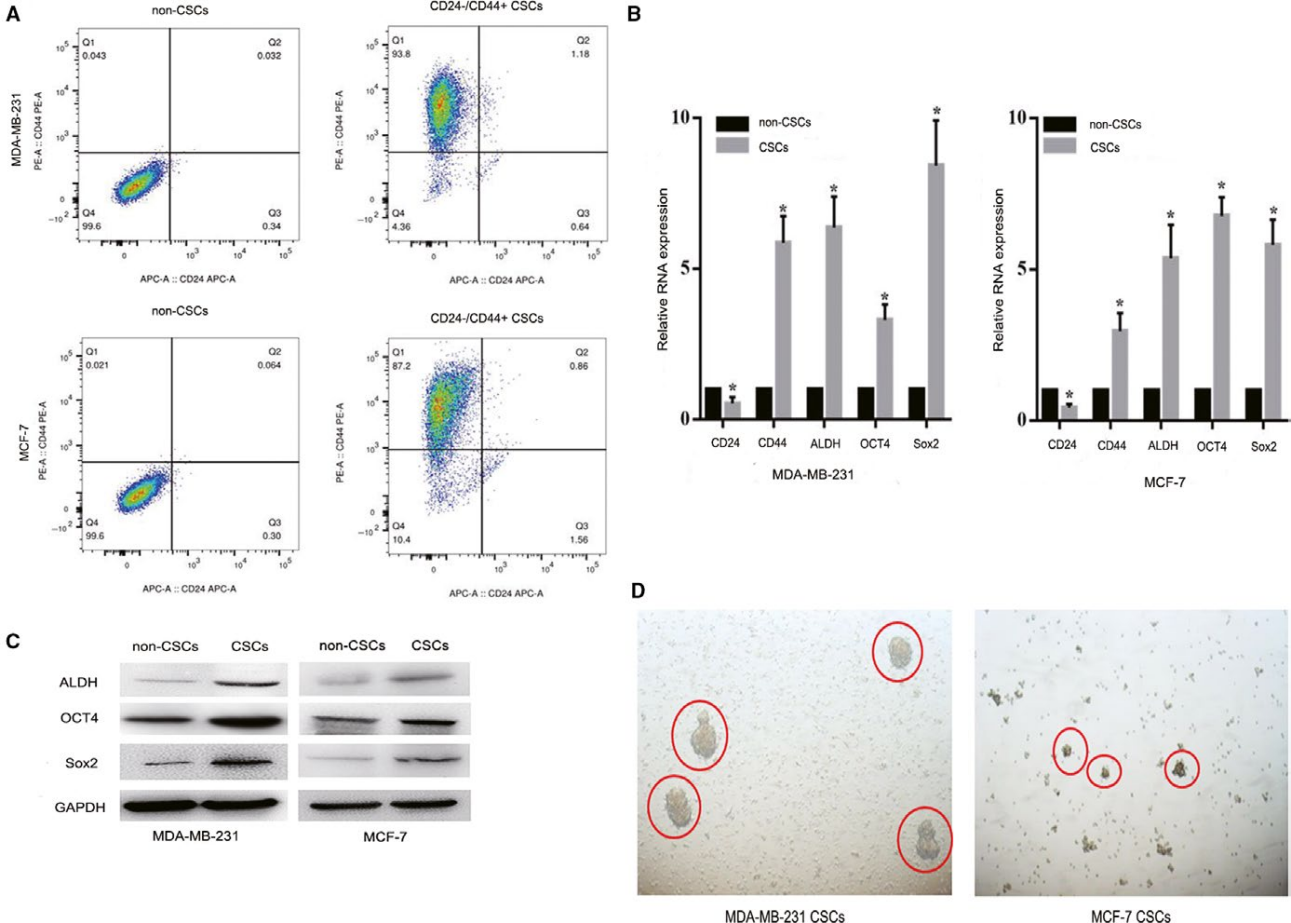
Isolation and characterization of BCSCs. A, Flow cytometry for analysis of CD24 and CD44 expression in two cell line CSCs (MDA‐MB‐231 and MCF‐7). B, qRT‐PCR analysis of CD24, CD44, ALDH1, Oct‐4, and Sox2 expression in all cell lines. C, The expression of ALDH1, Oct‐4, and Sox2 proteins was analyzed by western blot. D, Image of mammospheres generated by MDA‐MB‐231 and MCF‐7 CSCs at Day 7. Pictures were taken at 400× magnification

CD44, aldehyde dehydrogenase 1 (ALDH1), Oct‐4, and Sox2 have been widely accepted as important and reliable surface markers for the isolation of cancer stem cells in several cancers including breast cancer.^23^, ^24^ In order to analyze whether CD44^+^/CD24^−/low^ cells represented the CSC population of BCSC, the expressions of CD44 and CD24 were identified by flow cytometry. Then, several stem cell surface markers mentioned above were confirmed by qRT‐PCR and western blot, respectively. The results of flow cytometry assay showed that the proportion of CD44^+^/CD24^−/low^ represented the BCSC population were 93.8% and 87.2% in MDA‐MB‐231 and MCF‐7 CSCs, respectively (Figure 1A), indicating that the sorted cells established foundation for the next experiment. Then, we found that the expression levels of SOX2, Oct4, ALDH1, and CD44 were significantly higher in CD44^+^/CD24^−/low^ cells as compared to non‐CD44^+^/CD24^−/low^ in both MDA‐MB‐231 and MCF‐7 (all *P *<* *0.05), while CD24 was downregulated. MDA‐MB‐231 had a higher proportion of CD44^+^/CD24^−/low^ CSC ratio compared with the other subtypes (Figure 1B). Meanwhile, western blot results revealed that the protein expression of SOX2, Oct4, and ALDH1 was upregulated in MDA‐MB‐231 and MCF‐7 CD44^+^/CD24^−/low^ cells compared with non‐CSCs (Figure 1C). The results were reproduced in three independent experiments.

From the observation of plate state, it was found that single‐cell suspensions of CSC cells formed microsphere at the average of 7‐14 days in serum‐free medium. The ability to form spheroid bodies of CD44^+^/CD24^−/low^ cells were successfully cultured from TNBC cell lines compared with non‐TNBC cell line after 7‐10 days (Figure 1D). A small amount of spherical growth state can be noticed in TNBC cell lines after 7 days of culture, with good refraction and high density. After average of 10 days, mature mammospheres were formed with an increased density and size, a better refraction, and subcultured steadily. This phenomenon indicated that CD44^+^/CD24^−/low^ cells from TNBC cell lines exhibited the longest stability and self‐renewal than the other cell lines.

### 3.2 tDRs expression profiling between MDA‐MB‐231 and MCF‐7 CSCs

**Figure 2:**
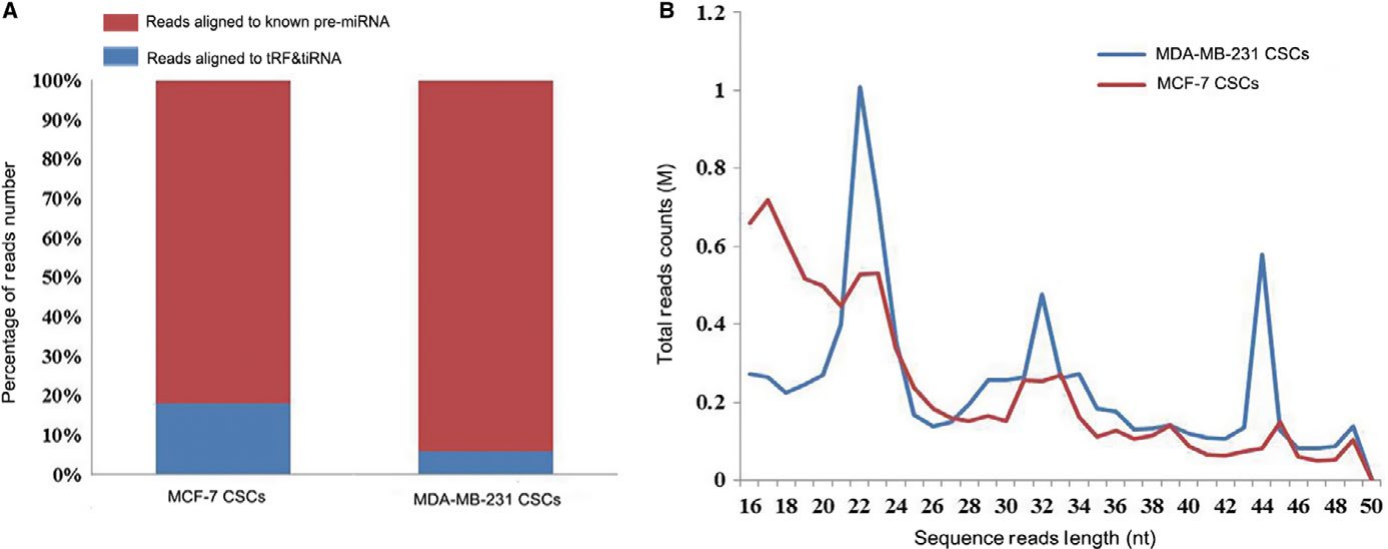
Summary of mapping results of aligned reads by MDA‐MB‐231 and MCF‐7 CSCs. A, The percentage of reads aligned to known pre‐miRNA and tRFs&tiRNAs. B, The sequence read length distribution of MDA‐MB‐231 and MCF‐7 CSCs

tRNA‐derived small noncoding RNAs expression profiles in non‐TNBC cell line MCF‐7 CSCs and TNBC cell line MDA‐MB‐231 CSCs were performed by RNA sequencing. A number of important informations can be obtained from these expression profiles. First, there were a total of 9 682 431 and 10 210 774 sequences from MCF‐7 and MDA‐MB‐231 CSCs, respectively, in our study. Secondary, a total of 173 003 and 781 604 sequence reads obtained from MCF‐7 CSCs that mapped to tRFs&tiRNAs and pre‐miRNA, respectively. Meanwhile, 90 026 and 1 427 970 sequences mapped to tRFs&tiRNAs and pre‐miRNA in MDA‐MB‐231 CSCs (Figure 2A). As expected, a substantial part of sncRNAs was from pre‐miRNAs, accounting for approximately 80%‐94% of total reads number. We speculated that the RNA quality was preserved during the RNA isolation from cell lines.

And then, the sequence read length distribution was showed in the line chart (Figure 2B). From the graphs, we can see that 51.75% and 67.85% of the tRNA‐derived reads align with pre‐tRNA or mature tRNA between 16 and 28 nucleotides from MDA‐MB‐231 and MCF‐7 CSCs, respectively, while the ratio of the sequencing reads derived from 5′ part or 3′ part of mature tRNA between 29 and 50 nucleotides was 48.25% and 32.15% in MDA‐MB‐231 and MCF‐7 CSCs, respectively.

### 3.3 Differential expression of tRFs&tiRNAs in MDA‐MB‐231 CSCs and MCF‐7 CSCs

**Figure 3:**
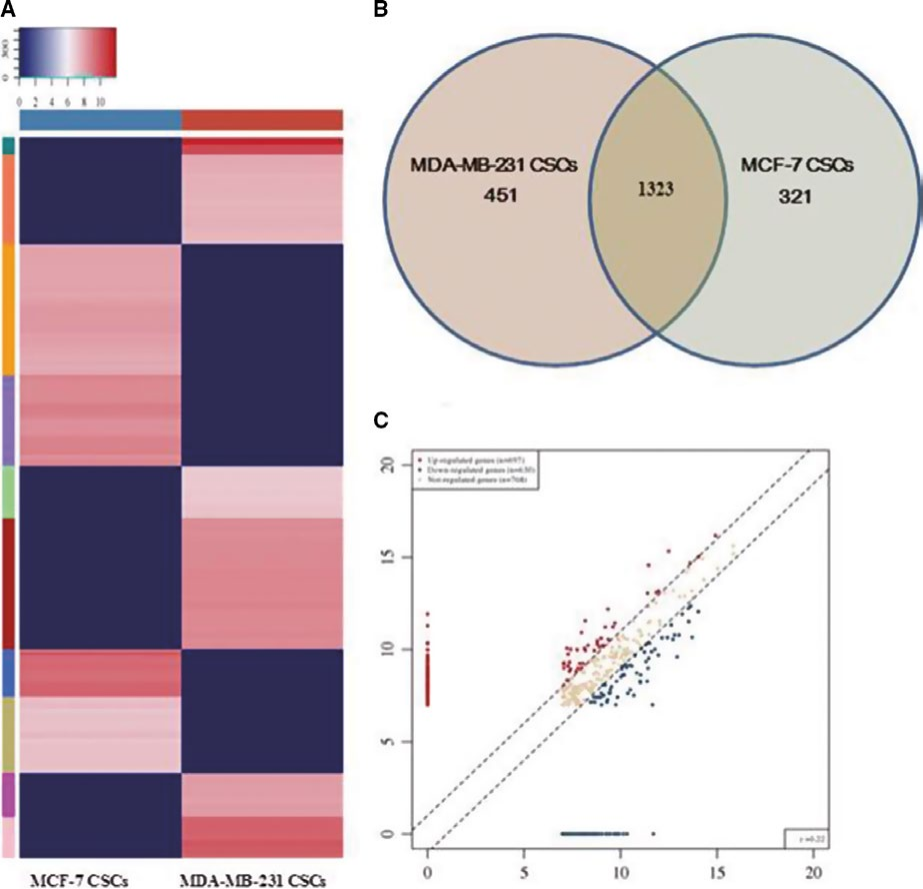
Differentially expressed tDRs between MDA‐MB‐231 and MCF‐7 CSCs. A, Heatmaps of differentially expressed tDRs in MDA‐MB‐231 and MCF‐7 CSCs. B, The gene number which expressed in the two groups and the specific expressed in each group. C, Scatter plot of differentially expressed tRFs&tiRNAs: Genes above the top line showed upregulation, and genes below the bottom line represented downregulation; gray dots indicated genes without differentially expression

Heat map of gene expression data obtained from non‐TNBC and TNBC groups (Figure 3A). A total of 321 and 451 specific expressed genes existed in two groups, respectively (Figure 3B). Principal component analysis (PCA) was performed to reduce the complexity of the data and deeply excavated the relationship and variation of the samples (Figure S1A). According to the expression level of each sample, calculated the correlation coefficient between all the samples (Figure S1B). The tRFs&tiRNAs expression variation between two compared groups with TPM values was shown in the scatter plot (Figure 3C).

Based on the results of RNA‐sequencing analysis, a total of 72 tDRs in MDA‐MB‐231 CSCs were statistically analyzed with the criteria fold change >2 and significant difference (*P *<* *0.05). It contained 18 upregulated and 54 downregulated tDRs in TNBC CSC group, comparing with the non‐TNBC group (Table S2). Detailed information about upregulated and downregulated tDRs was listed in Table S3. Among them, three tDRs exhibited a more than 10‐fold increase, 10 more than 10‐fold decrease, 4 and 11 tDRs more than fivefold increase and decrease, and three upregulated and 10 downregulated tDRs more than twofold change and accounted for 36.1% of all significantly differentially expressed tDRs.

### 3.4 Characterization of tRFs&tiRNAs derived from tRNAs

**Figure 4:**
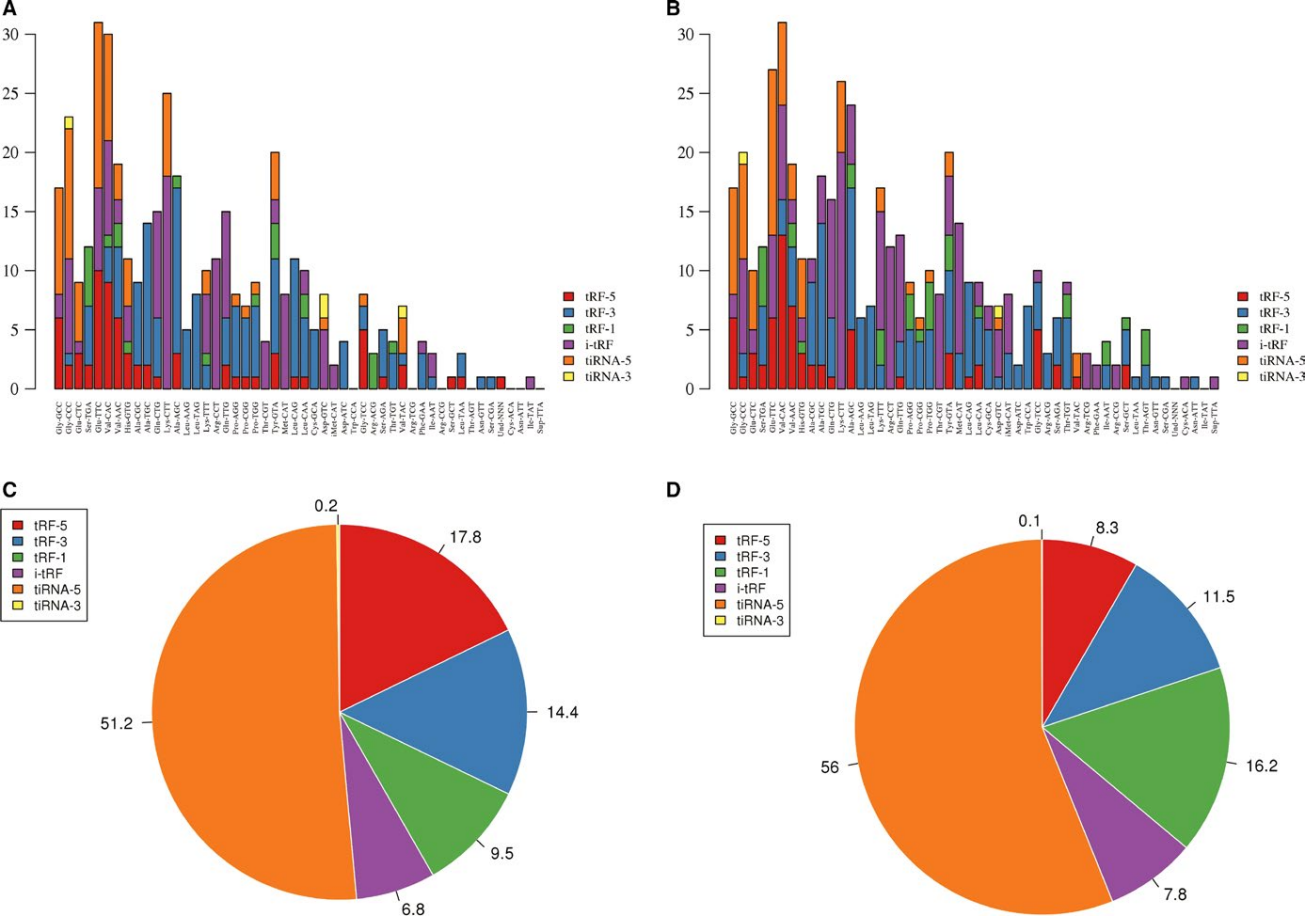
Characterization of tRFs&tiRNAs derived from tRNAs. A and B, Stacked plot for the number of tRFs&tiRNAs derived from the same anticodon of the tRNA: The X‐ and *Y*‐axis represented the tRNAs with the same anticodon tRNA and the number of different tRFs&tiRNAs derived from the same anticodon, respectively. C and D, The unique tRFs&tiRNAs of expressed level percentage of each subtype

The results show that 1327 tDRs were identified and mapped to 138 independent cytosolic tRNAs; some tDRs were mapped to more than one unique tRNA (Table S1). tRFs&tiRNAs with different sequence had the same anticodon or the same transfer amino acid. The stacked plot showed that the number of tRFs&tiRNAs derived from the same anticodon tRNA (Figure 4A,B). tRFs&tiRNAs are separated subtypes by their sites. From pie plot, we may discover that most of them derived from the 5′ parts of mature tRNAs and formed by a cleavage in the D loop, whereas tRFs and tiRNA‐3 series were limited to tRFs&tiRNAs. This pie plot was used to show the expression level (TPM values) percentage of each subtype of tRFs&tiRNAs in two groups (Figure 4C,D).

### 3.5 Verification of the selected tDRs levels in other stem cell lines and serum by quantitative real‐time PCR

**Figure 5:**
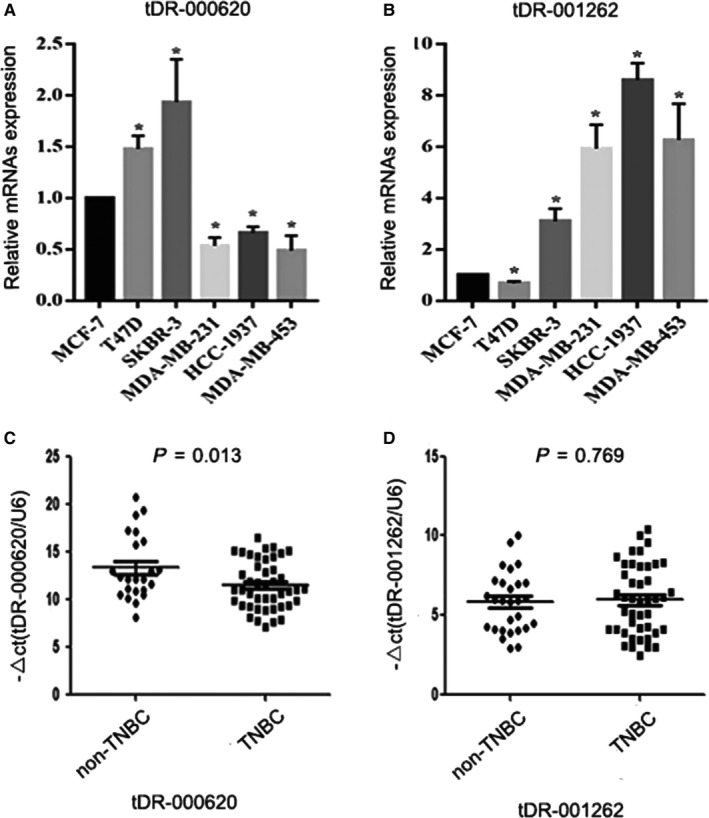
Validation of six dysregulated tDRs in triple‐negative breast cancer (TNBC) and non‐TNBC stem cell lines and serum samples. A and B, The expression level of tDR‐000620 and tDR‐001262 in HCC‐1937, MDA‐MB‐453 CSCs, and T47D, SKBR3 CSCs by qRT‐PCR appeared to be consistent with the result in MDA‐MB‐231 CSC group compared with the MCF‐7 CSC group. C, tDR‐000620 was significantly downregulated in serum of TNBC patients compared with non‐TNBC. D, The serum expression level of tDR‐001262 between two groups was not statistically significant; tDR‐001267, tDR‐008406, tDR‐000106, and tDR‐007257 were not detected in serum samples. (*P <* *0.05, n = 3)

To further verify the differentially expressed tDRs level from sequencing results, we selected six differentially expressed tDRs (tDR‐000620, tDR‐001262, tDR‐001267, tDR‐008406, tDR‐000106, and tDR‐007257) using qRT‐PCR in TNBC CSCs (HCC‐1937, MDA‐MB‐453, MDA‐MB‐231) and non‐TNBC CSCs (T47D, SKBR3, MCF‐7). The results showed that tDR‐000620 was significantly downregulated and tDR‐001262 was significantly upregulated in TNBC CSCs (all *P *<* *0.05 Figure 5A,B). However, tDR‐001267, tDR‐008406, tDR‐000106, and tDR‐007257 were not detected in all stem cell lines. Briefly, the expression patterns of the selected tDRs appeared to be consistent with the result in MDA‐MB‐231 CSC group compared with the MCF‐7 CSC group. This result was in concordance with the sequencing results, indicating that the sequencing result was reliable and good repeatability.

And then, we examined the six differentially expressed tDRs in the serum samples from 44 TNBC patients and 28 non‐TNBC patients. The scatter plot was used to show the relative expression of serum tDRs. From the scatter plot, we found that tDR‐000620 level was significantly downregulated in the triple‐negative subgroup (*t *=* *2.597, *P *=* *0.013, Figure 5C). But, we could not detect that the expression of tDR‐001262 had the same expression trend in serum from TNBC patients (*t *=* *0.295, *P *=* *0.769, Figure 5D). Similarly, tDR‐001267, tDR‐008406, tDR‐000106, and tDR‐007257 were not detected in serum samples.

### 3.6 Gene ontology and signaling pathway analysis of differently expressed tDRs

**Figure 6:**
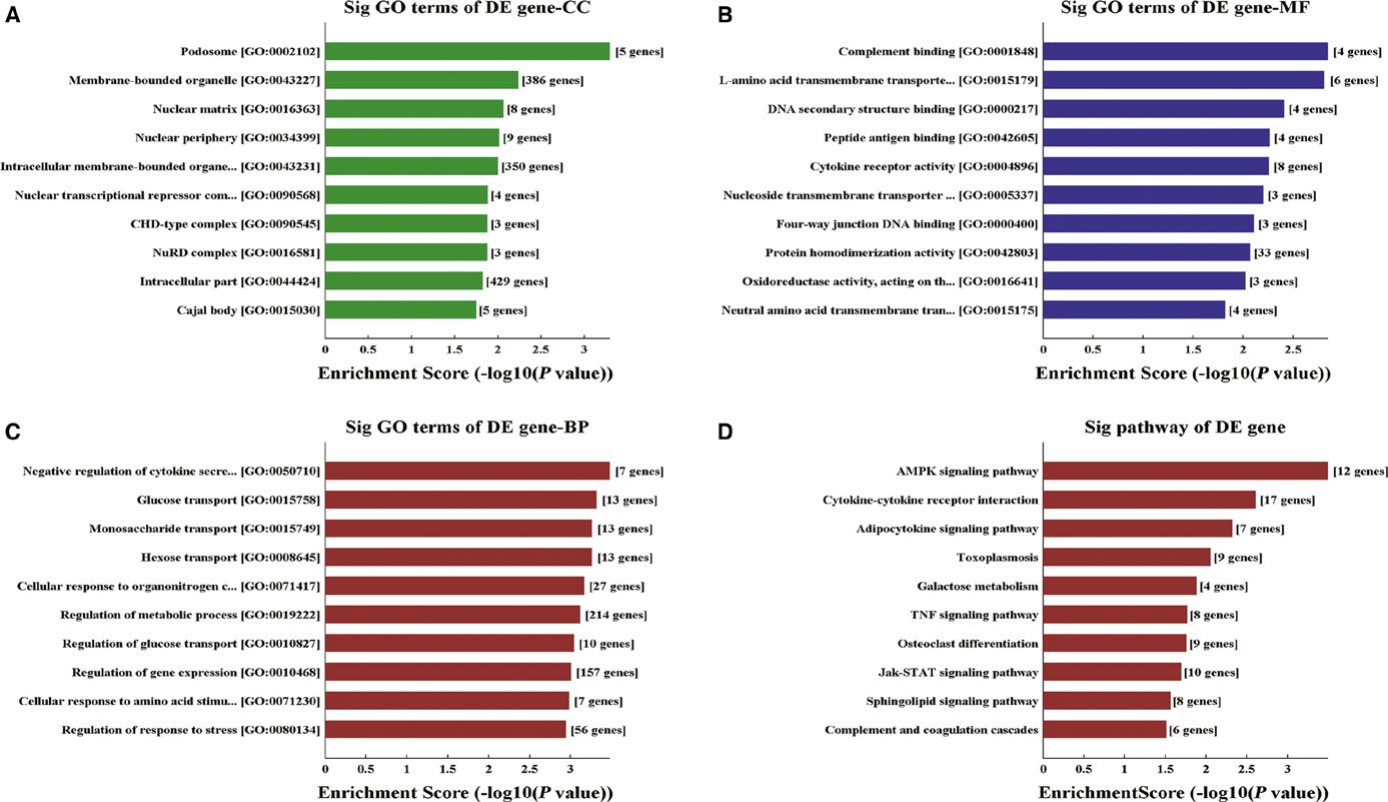
Gene ontology (GO) and KEGG analyses for target genes of tDR‐000620. A‐C, The mainly molecular function, biological process, and cellular component of target genes with fold changes >2. D, The pathway analysis indicated that these genes were involved in the cytokine–cytokine receptor interaction, AMPK signaling pathways, adipocytokine signaling pathways, toxoplasmosis, galactose metabolism, TNF signaling pathways, osteoclast differentiation, Jak‐STAT signaling pathways

GO analysis showed that most of gene products from tDR‐000620 (derived from tRNA^Glu‐TTC−8‐1^) were mainly located in intracellular part and membrane‐bounded organelle (Figure 6A). While a small percentage of these gene products were found in Nuclear periphery, Nuclear matrix, podosome, and so on. Meanwhile, the data showed that molecular functions of the target gene from tDR‐000620 mainly included protein homodimerization activity and cytokine receptor activity (Figure 6B). Furthermore, the genes functions were involved in the biological processes of metabolic, gene expression, and response to press (Figure 6C). To further investigate these gene products participated in signaling pathways, KEGG analysis was performed. The result showed that target genes are related to 10 signaling pathways. Cytokine‐cytokine receptor interaction and AMPK signaling pathways were the most significantly enriched pathways (Figure 6D). Meanwhile, the pathway analysis showed that these gene products also participated in other signaling pathways, involving adipocytokine signaling pathways, toxoplasmosis, galactose metabolism, TNF signaling pathways, osteoclast differentiation, Jak‐STAT signaling pathways, sphingolipid signaling pathways, and complement and coagulation cascades.

### 3.7 Serum tDR‐000620 level and clinicopathological features of TNBC patients

In total, 44 TNBC patients were enrolled in our cohort study. We validated the serum levels of tDR‐000620 in 44 TNBC patients by qRT‐PCR and investigated the relationship between tDR‐000620 expression and clinicopathological characteristics. According to median expression level as the cutoff point, patients were categorized into high expression group and low expression group. As shown in Table 2, the significant association was presented between tDR‐000620 expression with age (*P *=* *0.018), node status (*P *=* *0.026), and local recurrence (*P *=* *0.019) in TNBC. There was no significant relationship between tDR‐000620 expression and the other clinicopathological characteristics (*P *>* *0.05).

**Table 2:** Correlation of serum levels of tDR‐000620 with different clinicopathological factors in triple‐negative breast cancer patients

Characteristics	No. of patients (%)	tDR‐000620	Expression	*P*
		Low	High	
Age (y)				
<50	20 (45.5%)	7	13	0.018^a^
≥50	24 (54.5%)	17	7	
Histological grade				
I + II	23 (52.3%)	11	12	0.349
III	21 (47.7%)	13	8	
Menstruation				
Negative	24 (54.5%)	14	10	0.580
Positive	20 (45.5%)	10	10	
Tumor size				
<2 cm	4 (9.1%)	2	2	0.848
≥2 cm	40 (90.9%)	22	18	
Ki67 status				
<20%	2 (4.5%)	1	1	0.552
≥20%	42 (95.5%)	23	19	
Vascular invasion				
Negative	16 (63.6%)	6	10	0.086
Positive	28 (36.4%)	18	10	
Resection margin				
Negative	36 (81.8%)	19	17	0.617
Positive	8 (18.2%)	5	3	
Lymph node status				
Negative	25 (56.8%)	10	15	0.026^a^
Positive	19 (43.2%)	14	5	
Local recurrence				
Negative	32 (72.7%)	14	18	0.019^a^
Positive	12 (27.3%)	10	2	
Distant metastases				
Negative	36 (86.4%)	18	18	0.199
Positive	8 (13.6%)	6	2	

a *P* value <0.05 was regarded statistically significant. *P* values were calculated using the Pearson chi‐squared test and Fisher's exact test.

### 3.8 tDR‐000620 acts an independent factor for prediction of recurrence in TNBC patients

**Figure 7:**
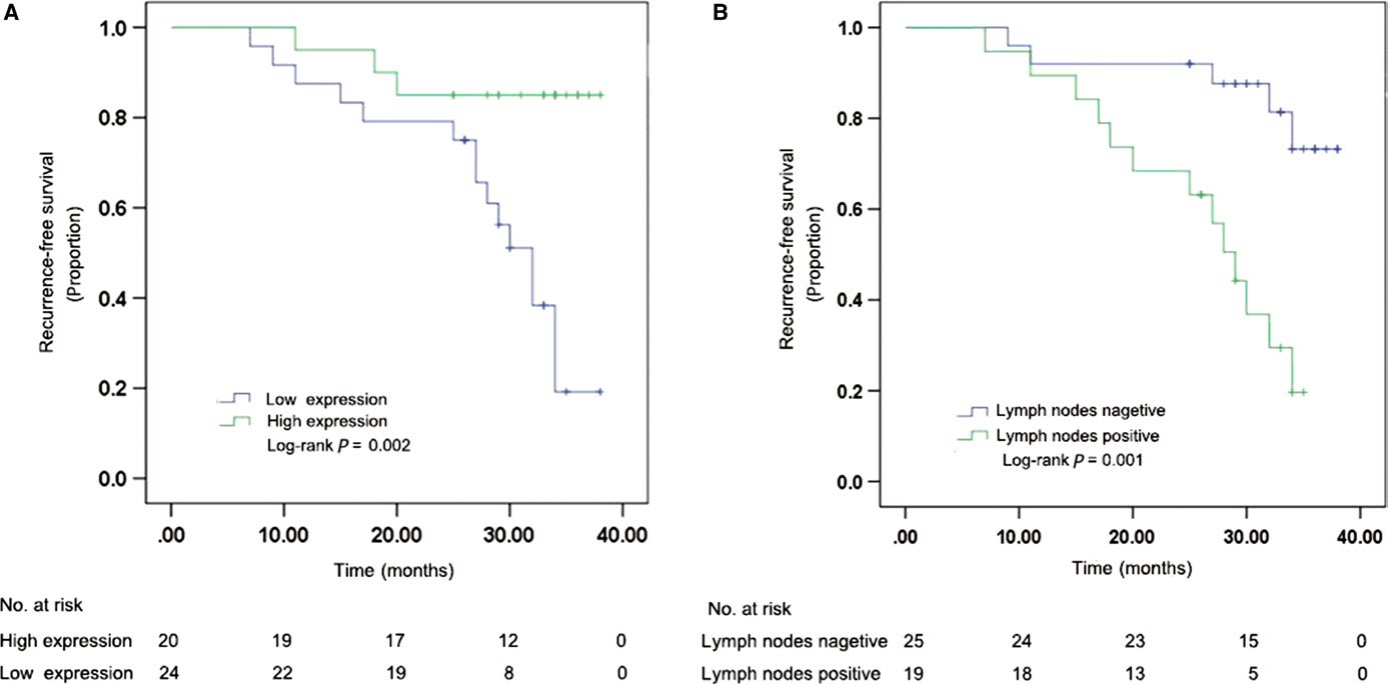
Comparison of recurrence‐free survival in tDR‐000620 and node status group of the triple‐negative breast cancer (TNBC) patients using Kaplan‐Meier method with log‐rank test. A, TNBC patients with low tDR‐000620 expression had significantly shorter recurrence‐free survival than those in high expression group. B, TNBC patients with lymphatic metastasis group had significantly shorter recurrence‐free survival than those in lymph node negative group

To compare the recurrence‐free survival of the TNBC patients with clinicopathological characteristics, Kaplan‐Meier method was used to calculate recurrence and metastasis rate and log‐rank test was carried out to compare each group. Local recurrence and distance metastasis are known collectively as recurrence. Of 44 patients, overall local recurrence and metastases occurred in 18 patients (40.9%) during follow‐up period. We further found that tDR‐000620 (*P *=* *0.002, Figure 7A) and node status (*P *=* *0.001, Figure 7B) showed significantly difference with recurrence‐free survival in TNBC patients. We further found that TNBC patients with tDR‐000620 low expression (5/24, 20.8%, Table 3) seemed to show a significantly high recurrence rate than patients with high expression (3/20, 15.0%, Table 3) within the first 2 years during follow‐up period. Meanwhile, TNBC patients with lymphatic metastasis showed a high risk of recurrence than lymph node negative (13/19, 31.6% and 5/25, 8.0%, Table 3). On the basis of log‐rank statistical analysis, multivariate Cox regression analysis was carried out to further investigate whether tDR‐000620 expression was an independent risk factor for recurrence in TNBC patients. As shown in Table 4, patients with low tDR‐000620 expression were an independent risk factor for overall recurrence and occurred recurrence earlier than patients with high expression of tDR‐000620 in TNBC (HR, 0.265; 95% CI, 0.073‐0.959; *P *=* *0.043; Table 4). As well, lymphatic metastasis was also found to be a significant independent adverse prognostic factor for recurrence‐free survival (HR, 3.616; 95% CI, 1.234‐10.596; *P *=* *0.019; Table 4). As stated above, these data indicated that low tDR‐000620 expression was identified as an independent adverse predictor of recurrence‐free survival in TNBC patients. As a result of short follow‐up time and less death‐related events, we cannot immediately conclude that the correlation between tDR‐000620 expressions with overall survival (OS).

**Table 3:** Correlation of different clinicopathological factors with recurrence in triple‐negative breast cancer patients

Characteristics	n	No of events	One‐year recurrence rate (%)	Two‐year recurrence rate (%)	*P*
Age (y)					
<50	20	6	15.0	20.0	0.185
≥50	24	12	4.2	16.8	
Histological grade					
I + II	23	8	0.0	4.3	0.135
III	21	10	19.0	33.3	
Menstruation					
Negative	24	8	4.2	8.3	0.237
Positive	20	10	15.0	30.0	
Tumor size					
<2 cm	4	1	0.0	0.0	0.403
≥2 cm	40	17	10.0	20.0	
Ki67 status					
<20%	2	1	0.0	0.0	0.866
≥20%	42	17	9.5	19.0	
Vascular invasion					
Negative	16	5	12.5	18.7	0.330
Positive	28	13	7.1	17.9	
Resection margin					
Negative	33	12	6.1	15.2	0.242
Positive	11	6	18.2	27.3	
Lymph node status					
Negative	25	5	8.0	8.0	0.001***
Positive	19	13	10.5	31.6	
tDR‐000620					
Low	24	15	12.5	20.8	0.002**
High	20	3	5.0	15.0	

**P value ≤0.01; ***P ≤* *0.001. *P* values were calculated using the log‐rank test.

**Table 4:** Multivariate Cox regression analyses of recurrence‐free survival in triple‐negative breast cancer patients

Variables	*B*	SE	Wald	*P*	HR	HR 95% CI
Node status	1.285	0.548	5.493	0.019^a^	3.616	1.234‐10.596
tDR‐000620 expression	−1.328	0.656	4.097	0.043^a^	0.265	0.073‐0.959

CI, confidence interval; HR, hazard ratio.

a *P* value <0.05 was considered statistically significant. *P* values were calculated using multivariate Cox proportional hazard model.

## 4 DISCUSSION

Triple‐negative breast cancer is a molecular subtype of high heterogeneity compared with the hormonal receptor positive and HER2 overexpressed breast cancers. TNBC cannot benefit from endocrine therapy and HER2‐targeted agents. Previous experimental and clinical evidence indicated that BCSCs may be the root of the recurrence, metastasis, and poor prognoses of TNBC patients. Deome et al^25^ demonstrated the first conclusive evidence of mammary stem cells in 1959. A population of CD44^+^/CD24^−/low^ cells from breast cancer patients has been isolated and demonstrated to have tumor‐initiating and sustainability in 2003.^8^ Subsequently, a series of studies confirmed the presence of CSCs was in breast cancer.^26^, ^27^ CD44^+^/CD24^−/low^, aldehyde dehydrogenase 1 (ALDH1), Oct‐4, and Sox2 have been widely accepted as important and reliable surface markers for the isolation and identification of BCSC.^23^, ^24^, ^28^, ^29^ Initially, we isolated CD44^+^/CD24^−/low^ cell subpopulation by MACS and analyzed stem cell characteristics in vitro. We found that CD44^+^/CD24^−/low^ cells represented the CSC population had a high proportion and the higher capacity of forming mammoshperes in TNBC CSCs compared with those in non‐TNBC. This phenomenon is consistent with previous studies.^8^, ^30^, ^31^ Furthermore, qRT‐PCR and western blot assays showed that the expression of ALDH1, Oct‐4, and Sox2 was upregulated with statistical significance in the CD44^+^/CD24^−/low^ cell subpopulation, particularly in TNBC. Flow cytometry revealed that the proportion of CD44^+^/CD24^−/low^ were 93.8% and 87.2% in MDA‐MB‐231 and MCF‐7 CSCs, respectively. While, some studies reported that CD44^+^/CD24^−/low^ and ALDH1 + are more enriched in TNBC tumor tissues compared with the other breast cancer subtypes.^32^, ^33^ The CD44^+^/CD24^−/low^ CSC ratio was an independent prognostic factor and may serve as a potential predictive marker for chemotherapy in breast cancer.^34^ Our observation coincides with the results of mentioned above. This phenomenon could be one reason of aggressive pathology in TNBC. Our study might contribute to better understanding tumor‐initiating and renewal capability of BCSCs related to the pathogenesis of TNBC.

With the spreading development of high‐throughput sequencing technology in recent years, tDRs have become a rising star in the regulation of biological processes and gene expression. Actually, many studies identified that abnormal expression of tRFs&tiRNAs is often participated in the pathogenesis of some diseases and served as potential diagnostic biomarkers or therapeutic targets.^14^, ^15^ However, only a limited number of studies have been evaluated the relation of tDRs and breast cancer.^16^ Yet now, there is still no information about the role of tRFs&tiRNAs in TNBC CSCs. In this study, our intention is to improve the understanding of tRFs&tiRNAs expression pattern in TNBC CSCs. High‐throughput sequencing technology was used to identify the differences in tDRs expression between TNBC cell line MDA‐MB‐231 CSCs and non‐TNBC cell line MCF‐7 CSCs. The sequencing data showed that tiRNAs were greater than tRFs in two cell lines. Among these tiRNAs, most of them belonged to the 5′‐halves (tiRNA‐5) series, whereas tiRNA‐3 series and tRFs were scarce. These results indicated that tRNA halves are produced by specific cleavage in the anticodon loop more than cleaved at one site and at varying rates in cells. Previous research indicated that 5′‐halves as intracellular molecules interacting with components of the translation initiation complex.^35^, ^36^ Therefore, we reason that tiRNA‐5 may be controlled in a cell‐specific manner and could even be produced through distinct mechanisms. These results indicated that the aggressive behaviors of TNBC may be regulated by these differentially expressed tRFs&tiRNAs from CSCs.

Furthermore, a total of 72 candidate tDRs which exhibited high counts and significantly differentially expressed between two CSC groups. 18 upregulated and 54 downregulated in TNBC group were detected. tDR‐000620, tDR‐001262, tDR‐001267, tDR‐008406, tDR‐000106, and tDR‐007257 were selected for validation using RT‐qPCR. tDR‐000620 and tDR‐001262 derived from tRNA^Glu‐TTC−8‐1^, and tRNA^Glu‐CTC−2‐1^ demonstrated stable and significantly differentially expressed in all stem cell lines which were further analyzed in serum samples. The expression pattern of tDR‐000620 in serum appeared accordance with the results CSCs. However, there were some different tDRs expression patterns between the CSCs and serum samples. Considering that it may be related to case characteristics, detection methods, and evaluation criteria.

Finally, tDR‐000620 differentially expressed in TNBC patient serum samples demonstrated that tDR‐000620 expression was correlated with age, node status, and recurrence. There was no correlation between tDR‐000620 expression and tumor size, stage, Ki‐67 status, and the other clinicopathological characteristics. At present, only one study revealed that 5′tRNA halves existed in blood with a stable form and their serum levels were regulated by age, either increase or decrease.^37^ Meanwhile, it is well known that TNBC is characterized by high risk of recurrence and metastasis. Considering this problem, we further investigated the association between clinicopathological features and recurrence‐free survival with Kaplan‐Meier method and log‐rank test. As shown in Table 3, tDR‐000620 and node status showed significantly difference with recurrence‐free survival in TNBC. Multivariate Cox regression analysis showed that lymphatic metastasis and low tDR‐000620 expression were two independent adverse predictive factors for recurrence‐free survival, which indicated that the patients in low tDR‐000620 expression and lymphatic metastasis group had significantly shorter recurrence‐free survival than those in high expression and lymph node negative group of TNBC. Given all that, we speculated that expression of tDRs‐000620 might be one possible reason of recurrence and poor prognosis and could indicate malignant biological behavior of TNBC. This finding establishes tDR‐000620 as a novel candidate biomarker for the early detection of recurrence in TNBC patients. A larger group of samples is needed to confirm our results. The potential mechanisms underlying the prognostic value of tDR‐000620 still need to be further explored.

The abundance of tDRs existed in different cell lines, and tissues have been implicated in biological processes and some diseases. Gene ontology (GO) and pathway analysis were performed to analyze the potential functions of these dysregulated tDRs. Most target genes of tDR‐000620 which belonged to tiRNA‐5 series in TNBC were associated with biological processes of metabolic, gene expression, and response to press. Hence, we inferred that the aggressive behaviors of TNBC may be related to these biological processes. Coincidentally, some tRNA halves were produced via the cleavage of mature tRNAs under stress stimulation, such as oxidative stress, UV irradiation, and the others.^38^ Therefore, tRNA halves were also known as tRNA‐derived stress‐induced RNAs. This may provide new evidences for understanding that tiRNAs are not random degradation fragments, but represent a novel class of small RNAs by a variety of stress stimulation. And furthermore, a majority of dysregulated gene products were involved in cytokine‐cytokine receptor interaction and AMPK signaling pathways. AMPK signaling pathway has been previously shown to inhibit mTOR and impair cell cycle progression at S and G2/M phases, playing a significant role in BCSC by Lee et al.^39^ Meanwhile, previous study also reported that there is a connection between BCSC and cytokine‐cytokine receptor interaction.^40^ With regard to the GO and KEGG pathway analysis presented, the relationship between these biological processes and TNBC CSCs should be well studied in the future.

In summary, we validated a range of differentially expressed tDRs between TNBC and non‐TNBC CSCs. Furthermore, we also identified tDR‐000620 may serve as an independent adverse prognostic factor of recurrence‐free survival in TNBC. These findings may provide new insight to study therapeutic strategies for eradicating the tumorigenic subpopulation of stem cells in TNBC. However, it is necessary to elucidate function and undergoing molecular mechanism of tDR in TNBC CSCs. Future research should expand the sample size and validate the relationship between tDR and cancer stem cell properties of TNBC.

## CONFLICT OF INTEREST

We declare that there is no conflict of interests.
