# Supplementary figures and images for: Identification of tRNA‐derived small noncoding RNAs as potential biomarkers for prediction of recurrence in triple‐negative breast cancer
Source: Cancer Med. 2018 Sep 21;7(10):5130–44. doi: 10.1002/cam4.1761 (PMC6198211; doi:10.1002/cam4.1761)

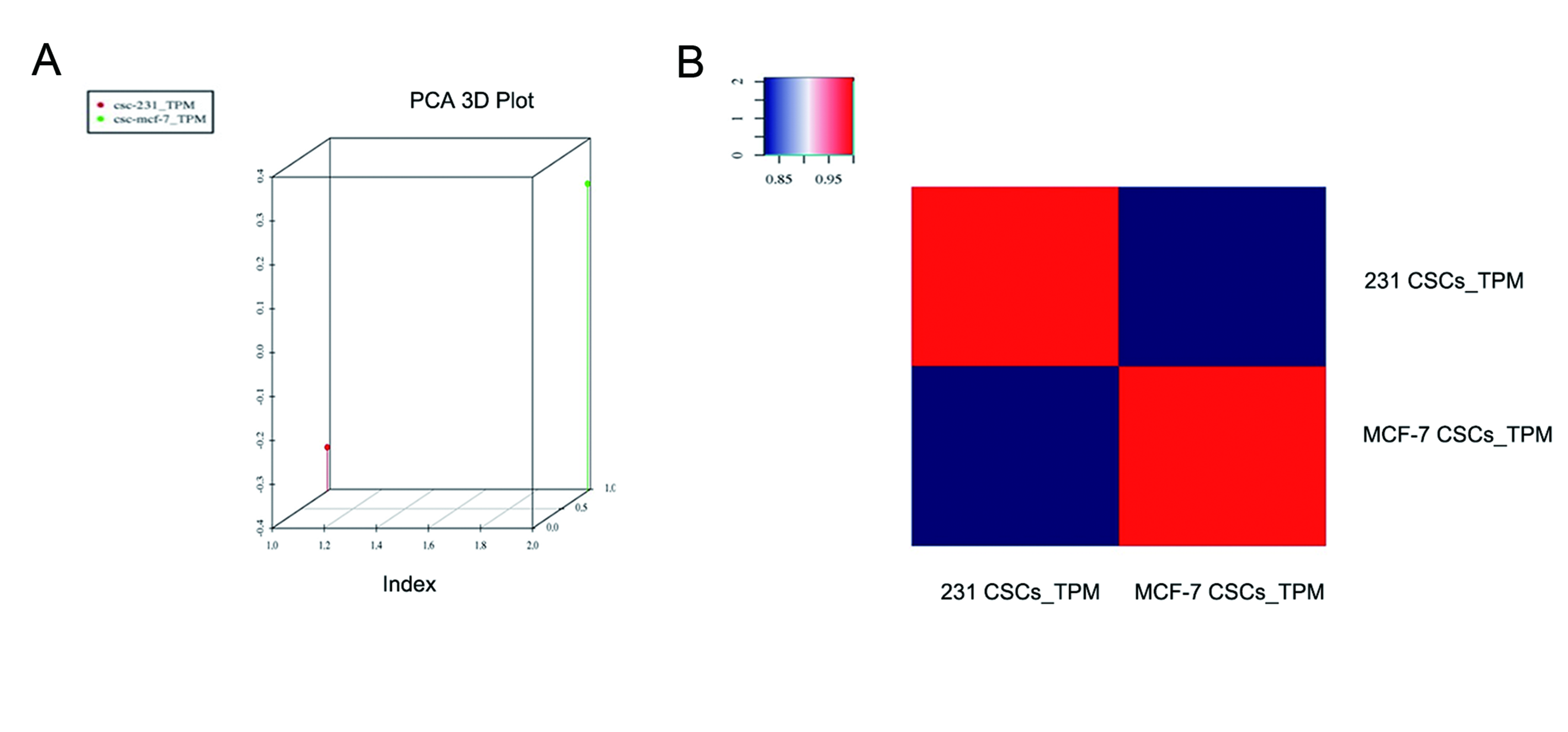

Supplement: Supplementary file 1 [file CAM4-7-5130-s001.tif]
